# High-sensitive cardiac troponin I (hs-cTnI) concentrations in newborns diagnosed with spinal muscular atrophy

**DOI:** 10.3389/fped.2023.1259293

**Published:** 2023-11-16

**Authors:** Jessika Johannsen, Deike Weiss, Joenna Driemeyer, Jakob Olfe, Fridrike Stute, Ferdinand Müller, Marion Schütt, Regina Trollmann, Heike Kölbel, Ulrike Schara-Schmidt, Janbernd Kirschner, Astrid Pechmann, Astrid Blaschek, Veronka Horber, Jonas Denecke

**Affiliations:** ^1^ Department of Pediatrics, University Medical Center Hamburg-Eppendorf, Hamburg, Germany; ^2^ Clinic for Children’s Heart Medicine and Adult Congenital Heart Disease, University Heart and Vascular Center, University Medical Center Hamburg-Eppendorf, Hamburg, Germany; ^3^ Department of Medical Biometry and Epidemiology, University Medical Center Hamburg-Eppendorf, Hamburg, Germany; ^4^ Department of Neonatology and Pediatric Intensive Care Medicine, University Children's Hospital, University Medical Center Hamburg-Eppendorf, Hamburg, Germany; ^5^ Department of Pediatrics, Division of Pediatric Neurology, University Hospital Erlangen, Erlangen, Germany; ^6^ Department of Pediatric Neurology, Centre for Neuromuscular Disorders, Center for Translational Neuro and Behavioral Sciences, University of Duisburg-Essen, Duisburg, Germany; ^7^ Department of Neuropediatrics and Muscle Disorders, Medical Center—University of Freiburg, Faculty of Medicine, University of Freiburg, Freiburg, Germany; ^8^ Dr. v. Hauner Children’s Hospital, Department of Pediatric Neurology and Developmental Medicine, LMU Munich University Hospital, Munich, Germany; ^9^ Department of Paediatric Neurology, University Children’s Hospital Tübingen, Tübingen, Germany

**Keywords:** high-sensitive troponin 1, neonates, cardiotoxicity, gene replacement therapy, onasemnogene abeparvovec

## Abstract

**Background:**

Spinal muscular atrophy (SMA) is a genetic neurodegenerative disease leading to muscular weakness and premature death. Three therapeutic options are currently available including gene replacement therapy (GRT), which is potentially cardiotoxic. High-sensitive cardiac troponin I (hs-cTnI) is widely used to monitor potential cardiac contraindications or side effects of GRT, but reference data in healthy newborns are limited and lacking in neonates with SMA. The aim of this study is to determine the range of pre-therapeutic hs-cTnI concentrations in neonates with SMA and to provide guidance for the assessment of these values.

**Methods:**

Hs-cTnI levels, genetic and clinical data of 30 newborns (age range 2–26 days) with SMA were retrospectively collected from 6 German neuromuscular centers. In addition, hs-cTnI levels were measured in 16 neonates without SMA.

**Results:**

The median hs-cTnI concentration in neonates with SMA was 39.5 ng/L (range: 4–1205). In 16 newborns with SMA, hs-cTnI levels were above the test-specific upper reference limit (URL). Exploratory statistical analysis revealed no relevant correlation between hs-cTnI levels and gender, gestational age, mode of delivery, SMN2 copy number, symptoms of SMA or abnormal cardiac findings.

**Discussion:**

Our results suggest higher hs-cTnI plasma levels in newborns with and without SMA compared to assay-specific reference values generated in adults. Given the wide range of hs-cTnI values in neonates with SMA, hs-cTnI levels must be determined before treatment in each patient and post-treatment elevations should be interpreted in the context of the course rather than as individual values.

## 1. Introduction

5q-associated SMA is an autosomal recessive neuromuscular disease characterized by dysfunction and in later stages apoptosis of the lower motor neuron resulting in progressive muscular weakness and premature death. It is caused by homozygous deletion or mutation of the *SMN1* (survival motor neuron) gene resulting in SMN protein deficiency. Currently, three alternative therapeutic options are available namely nusinersen, onasemnogene abeparvovec and risdiplam. Since October 2021 SMA newborn screening has been implemented in Germany enabling the diagnosis and treatment of even asymptomatic newborns with SMA. Onasemnogene abeparvovec is a single dose, intravenous gene replacement therapy (GRT) providing a copy of the *SMN1* gene via an AAV9 vector (1). In clinical trials and post-marketing, elevation of cardiac enzymes potentially pointing towards cardiac toxicity has been observed after application of onasemnogene abeparvovec (2–5). Therefore, the determination of cardiac troponin I (cTnI) was recommended as a monitoring parameter, although there is no experience yet with the classification of these values in normal and abnormal, respectively, in infants with SMA (6). Moreover, SMA has been considered to be a multisystemic disease with cardiac manifestations as congenital heart disease and/or arrhythmias at least in severely affected patients (7) making a cardiologic work-up in SMA patients reasonable.

The troponin complex regulates the contraction in skeletal and cardiac muscle and consists of three proteins troponin I, troponin T and troponin C. Cardiac-specific troponin T (cTnT) and I (cTnI) are markers for ischemic and non-ischemic cardiac injury (8).

In clinical practice, the analysis of high-sensitive cTnI (hs-cTnI) is increasingly replacing the previously used determination of hs-cTnT values. Data reporting hs-cTnI levels are limited in healthy neonates and are completely lacking in newborns with SMA to date. However, because elevated cardiac troponin T levels have been observed in untreated type 1, 2, and 3 SMA patients without cardiac disease, information on hs-cTnI levels in neonates with SMA is especially important. Additionally, knowledge about pre-therapeutic and age-appropriate hs-cTnI levels in newborns with genetically confirmed SMA is relevant for the decision making for one of the three available therapeutic options, considering possible cardiotoxic effects of GRT.

Already healthy newborns had higher serum hs-cTnI concentrations (median 21.54 ng/L (95th percentile 139.36 ng/L) and 9.3 ng/L (75th percentile 93.8 ng/L), respectively) in two studies of 36 and 24 healthy newborns compared to healthy adults (9, 10). Since the approval of GRT, we have observed hs-cTnI levels in neonates with SMA that exceed reported hs-cTnI levels in healthy neonates. Further cardiologic diagnostics (echocardiography, ECG) in these patients showed no evidence of cardiac damage, so we interpreted these values as in the “normal” range for this specific disease and age group. To follow up on our observation, we retrospectively collected data on hs-cTnI serum concentrations in a multicenter cohort of 30 neonates with genetically proven 5q-SMA and aimed to describe their age-appropriate and pre-therapeutic hs-cTnI concentrations. We hypothesize that hs-cTnI levels in neonates with SMA may be above the test-specific URLs obtained in adults. Elevated hs-cTnI levels after treatment should then be interpreted in the context of the pre- and post-treatment course and not as single values.

## 2. Material and methods

### 2.1. Patients

Since October 2021, newborn screening for 5q-associated SMA has been implemented in clinical routine in Germany. Medical records of neonates that were diagnosed with genetically confirmed SMA after abnormal SMA newborn screening in the Departments of Pediatrics/Neuropediatrics of the participating University medical centers in Hamburg, Erlangen, Essen, Freiburg, Munich and Tübingen were retrospectively reviewed for the following: gender (male, female), gestational age (term, preterm), mode of delivery (caesarean section, vaginal delivery), APGAR score (abnormal was considered below 8), umbilical cord pH (abnormal was considered below 7,2), *SMN2* copy number, age at lab samplings, levels of hs-cTnI (ng/L), N-terminal pro-brain natriuretic peptide (NT-proBNP, in ng/L), creatinkinase (CK, in U/L), echocardiography and electrocardiogram. Additionally, motor function of the newborns assessed by the Children’s Hospital of Philadelphia Infant Test of Neuromuscular Disorders (CHOP INTEND) scale, use of assistant ventilation and the need for feeding support (nasogastric tube, gastrostomy) were collected. Only neonates in whom at least one hs-cTnI value was available in the first 28 days of life were included. Retrospective data collection was approved by the local ethics committee (2022-100987-BO-ff) in Hamburg. Parents or guardians consented to the regular diagnostic work-up.

### 2.2. Laboratory analysis

**Figure 1:**
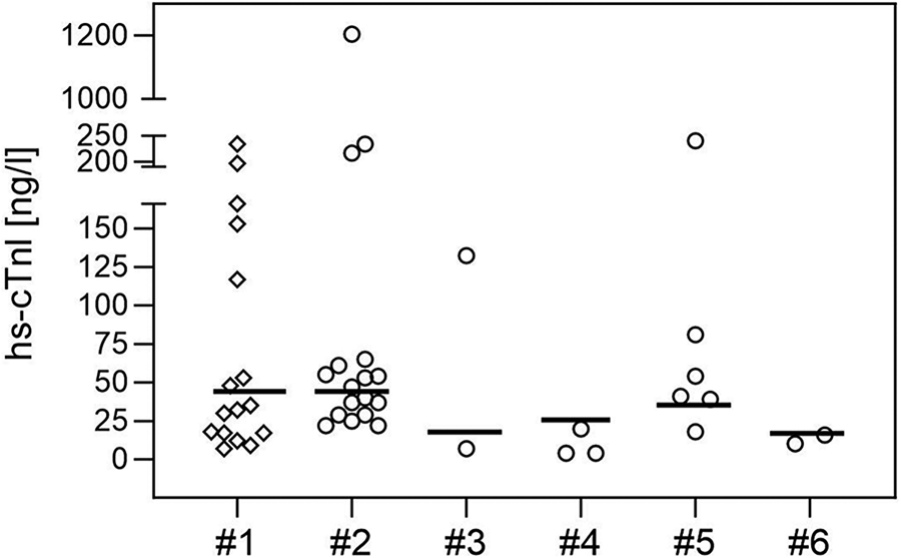
hs-cTnI concentrations (in ng/l) in 30 neonates with genetically confirmed SMA, shown individually for each assay used (circles, #2: Siemens/Atellica, #3 Beckman Coulter/Access, #4 Abbott Diagnostics/Architect, #5 Siemens/Centaur, #6 Abbott Diagnostics/Alinity). Hs-cTnI values of 16 neonates without SMA (measured with the Siemens Atellica immunoassay are shown in row #1 (rhombus). The bold lines mark the test-specific URLs.

Blood samples for routine parameters and cardiac enzymes were routinely collected in SMA patients in the initial process of confirming the genetic diagnosis and before treatment initiation. Hs-cTnI concentrations in SMA patients were measured using different commercial immunoassays (Siemens/Atellica (*n* = 17) or Centaur (*n* = 6), Abbott Diagnostics/Alinity (*n* = 2) or Architect (*n* = 3), Beckman Coulter/Access (*n* = 2)) with test-specific upper reference levels (URL) in each center (15.6–45 ng/L, Figure 1). We note the test-specific URL to provide context for the values from the SMA patients. However, test-specific URLs were derived from adult cohorts and were not provided as age-appropriate URLs.

### 2.3. Statistical analysis

All data were summarized using descriptive statistics. Categorical data are summarized by absolute and relative frequencies. Continuous data are summarized by median, mean, first and third quartile, minimum and maximum. Pearson’s chi-squared test and Fisher’s exact tests were performed for the exploratory assessment of the binary hs-cTnl (within vs. above the test-specific URL) regarding potential influences of binary variables gender, gestational age, abnormal APGAR score, abnormal umbilical cord pH, mode of delivery, abnormalities in echocardiography and/or electrocardiogram, SMA symptoms, NT-proBNP binary and CK binary. Spearman’s correlation coefficients were calculated for the exploratory assessment of the correlation between the continuous hs-cTnI and the continuous variables NT-proBNP levels, CK levels and CHOP INTEND. The Kruskal-Wallis test was performed to assess the categorical variable *SMN2* copy number regarding a difference in the continuous hs-cTnI. To investigate the difference in hs-cTnI regarding the binary age at laboratory testing (≤28 days, >28 days), a linear mixed model approach, with patient as random effect and binary age within patient as nested random effect was performed. The hs-cTnI was analyzed using a logarithmic transformation. In this exploratory data analysis all variables were compared and statistically assessed for descriptive purposes and not in a confirmatory sense. Therefore, *p*-values are not adjusted for multiple testing, but used as descriptive measures.

All analyses were conducted with SAS^©^ 9.4.

### 2.4. Sensitivity analysis

Since the upper reference values of the immunoassays were obtained in adults, hs-cTnI levels routinely taken during the diagnostic workup of newborns with mild, non-cardiac conditions and without perinatal complications were used as an orientation control group (Supplementary Table S1). Hs-cTnI levels were measured using the Siemens/Attelica immunoassay, as this assay was also used in the majority of SMA patients (*n* = 17).

## 3. Results

### 3.1. Patients' characteristics

During the first 13 months of the national SMA newborn screening, data were collected from 30 neonates (19 female, 11 male) with genetically confirmed 5q-associated SMA and at least one hs-cTnI value in the first 28 days of life in the participating University medical centers. Of these patients, one (3,3%) patient had 1 *SMN2* copy, 15 (50%) patients 2 *SMN2* copies, 9 (30%) patients 3 *SMN2* copies and 5 (16.7%) patients 4 *SMN2* copies. Fourteen patients were delivered via caesarean section and 25 patients were born at term. One patient had an APGAR score below 8 and in 3 neonates umbilical cord pH was below 7.2.

Four patients showed echocardiographic abnormalities including atrial septal defect type II, a combination of atrial septal defect (ASD) type 2 with left-to-right-shunt and small muscular ventricular septal defect (VSD) and partial anomalous pulmonary venous connection (PAPVC). Persistent foramen ovale was found in 9 neonates and classified as normal in context of the age. Three patients showed abnormal electrocardiography results namely prolonged QT time and ST segment depression in V1/V2.

Motor function assessed by CHOP INTEND showed a median score of 43 pts. (range: 2–62, mean: 39). Five patients had clinical SMA symptoms according to the evaluator and 25 patients were asymptomatic. Symptoms included muscular hypotonia, reduced head control or spontaneous movements, areflexia and/or tongue fasciculations. None of the neonates needed prolonged assistant ventilation and/or feeding support. One patient needed CPAP ventilation for 30 min after birth.

Demographic and clinical data are summarized in Table 1.

**Table 1:** Demographic and clinical characteristics of 30 newborns with SMA.

Gender, *n* (%)	
Male	11 (36,7)
Female	19 (63,3)
*SMN2* copy number, *n* (%)	
1	1 (3,3)
2	15 (50)
3	9 (30)
4	5 (16,7)
Mode of delivery, *n* (%)	
Caesarean section	14 (48,3)
Vaginal delivery	15 (51,7)
Unavailable data	1
Gestational age, *n* (%)	
Term	25 (83,3)
Late-preterm	5 (16,7)
Unavailable data	0
APGAR score < 8, *n* (%)	1 (3,4)
Unavailable data	1
UA-pH < 7,2, *n* (%)	3 (11.5)
Unavailable data	4
Assistant ventilation, *n* (%)	
Yes	0 (0)
No	30 (100)
Unavailable data	0
Feeding support, *n* (%)	
Yes	0 (0)
No	30 (100)
Unavailable data	0
Abnormal Echocardiography, *n* (%)	
Total	4 (14,8)
ASD II	2 (7,4)
ASD II and muscular VSD	1 (3,7)
PAPVC	1 (3,7)
Unavailable data	3
Abnormal ECG, *n* (%)	
Total, *n* (%)	3 (11,5)
Prolonged QTc	2 (7,7)
ST segment depression in V1/V2	1 (3,8)
Unavailable data	4
SMA symptoms, *n* (%)	
Yes	5 (16,7)
No	25 (83,3)
Unavailable data	0
CHOP INTEND score, (*n* = 29),	
Median (Q1, Q3, range)	43 (29, 54, 2–62)
Unavailable data (*n* = 1)	

### 3.2. Laboratory results in SMA patients

Across the cohort, first hs-cTnI values were obtained at the median age of 13 days (range 2–26, mean 14.2) with a median hs-cTnI concentration of 39.5 ng/L (range: 4–1205, mean: 96.63). In 16 (53%) of 30 neonates hs-cTnI value was above the test-specific URL (Table 2, Figure 1). Hs-cTnI concentrations were measured with the Siemens/Atellica immunoassay in 17 of the 30 newborns, and 9 (53%) had hs-cTnI levels above the test-specific URL given by the test provider. The wide range of hs-cTnI concentrations was due to high concentrations found in one patient with values of 1205 and 1526 ng/L at 17 and 26 days of age, respectively. This patient was born at term without complications (including normal APGAR score and umbilical-cord pH) and showed no clinical signs of SMA. However, echocardiography revealed an ASD type 2 with left-to-right-shunting and a small muscular VSD. NT-proBNP values were also elevated with 2310 ng/L and 2738 ng/L at the age of 17 and 26 days, respectively, but CK values were normal. The patient with PAPVC and ST segment depression in V1/V2 had a hs-cTnI value above the test-specific URL (241 ng/L, URL obtained in adults: 37 ng/L). Hs-cTnI values below the test-specific URL were found in the other patients with cardiac findings (ASD II and prolonged QT time, respectively). Only one of the patients with SMA symptoms had a hs-cTnI value slightly above the test-specific URL (40 ng/L, URL: 37 ng/L).

**Table 2:** hs-cTnI levels (ng/L), NT-proBNP levels (ng/L) and total CK levels (U/L) in our cohort of 30 newborns (≤28 days of age) with SMA.

	n^a^	Median	Mean	Q1, Q3	Range
hs-cTnI	30	39,5	96,63	22, 61	4–1205
NT-proBNP	17	1108	1178,4	833, 1375	291–2396
CK	28	105	123,9	68, 166	34–294

^a^ Number of patients in whom the parameter was available.

Results of NT-proBNP and CK are summarized in Table 2.

**Figure 2:**
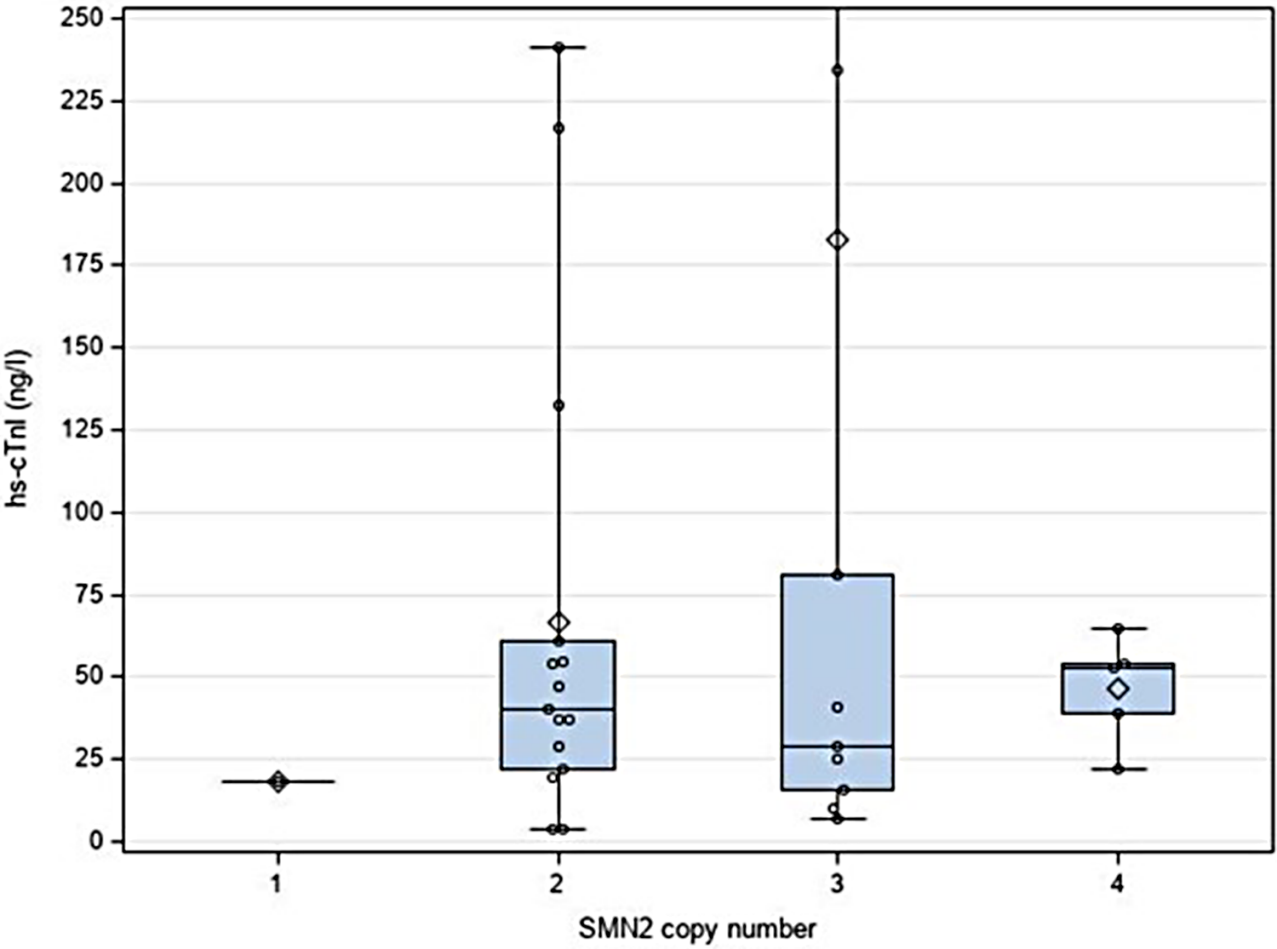
Correlation between hs-cTnI concentrations (in ng/l) and *SMN2* copy number. High hs-cTnI value (1205 ng/L) in one newborn with 3 *SMN2* copies number has not been included for clearer presentation. Median value is shown as a line; the rhombus represents the mean value.

**Figure 3:**
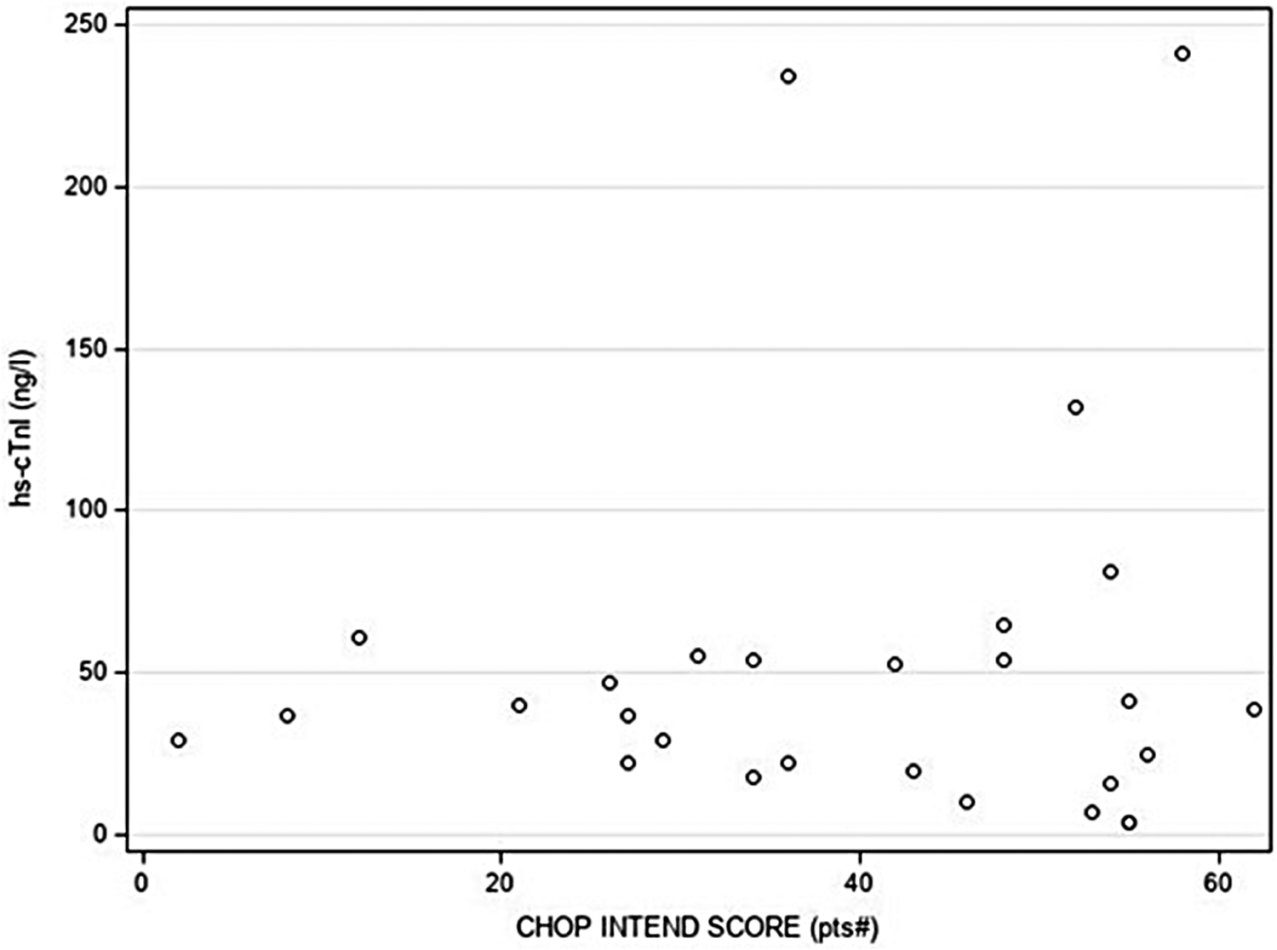
Correlation between hs-cTnI concentrations (in ng/l) and motor function score (measured by CHOP INTEND). Low CHOP INTEND score is indicating muscle weakness in SMA patients. One newborn with a high hs-cTnI value (1205 ng/L) and 44 points in CHOP INTEND has not been included for clearer presentation.

**Figure 4:**
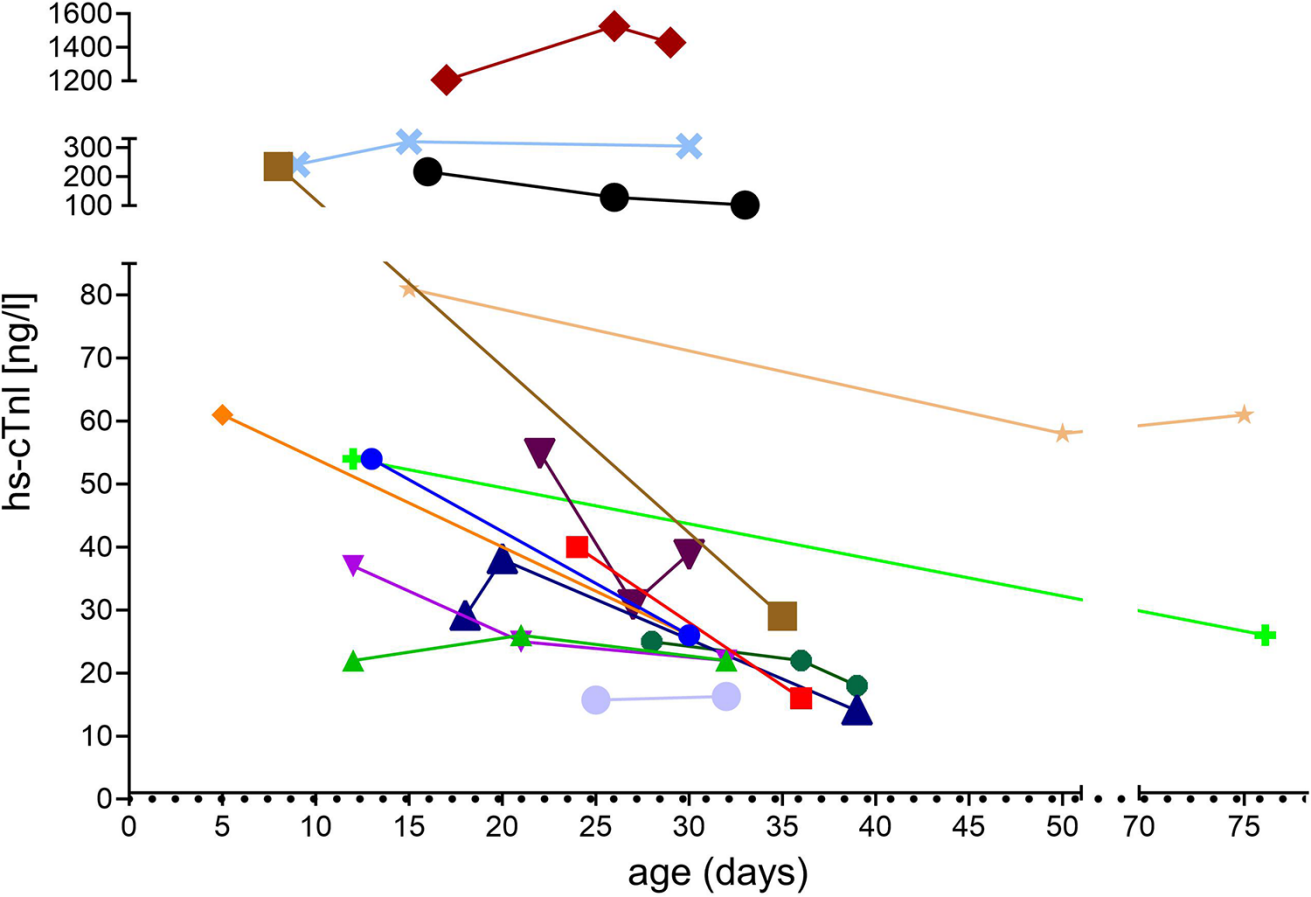
Course of hs-cTnI concentrations in 15 patients with SMA after the neonatal period.

Statistical analysis revealed no relevant association or correlation between hs-cTnI levels that were beyond the test-specific URL and any of the variables. In particular, *SMN2* copy number, clinical symptoms of SMA or abnormal cardiac findings were not associated with hs-cTnI levels above the test-specific URL (Figure 2). Additionally, no correlations between motor function score (measured by CHOP INTEND), levels of CK and NT-proBNP, respectively, and hs-cTnI concentrations were found (Figure 3). In 15 patients, another hs-cTnI value was available after the neonatal period (Figure 4). In 10 of these patients, the initial hs-cTnI in the neonatal period was higher than the URL, in 5 of these patients the value decreased below the URL in the follow-up period, and in 5 patients the value remained above the URL.

### 3.3. Hs-cTnI levels in newborns without SMA or cardiac disease

hs-cTnI levels were measured in 16 newborns (age at test: 0–5 days) as part of the diagnostic work-up for suspected but not confirmed neonatal infection or metabolic disease, hyperbilirubinemia, prenatal cerebral hemorrhage, or maternal disease (medication, infection, Basedow’s disease). Hs-cTnI values were within the test-specific URL (median: 33.5 ng/L; range: 7–234 ng/L, mean: 71.6) in 9 neonates. In 7 (44%) newborns, hs-cTnI concentrations were above the test-specific URL (Figure 1).

## 4. Discussion

High-sensitive cardiac troponin I has been established as a sensitive and specific marker for cardiac injury in adults and children (11) but reference data are still limited in healthy newborns and missing in newborns with SMA, respectively. Considering possible cardiotoxic side effects of recently implemented therapies for SMA, e.g., GRT, potential risk factors must be clarified before deciding on this treatment. In this context, the evaluation of high-sensitive cardiac Troponin I (hs-cTnI) values is used, which is also increasingly replacing the earlier analysis of hs-cTnT in clinical practice. Thus, we retrospectively evaluated pre-therapeutic hs-cTnI levels that were routinely assessed in 30 newborns with SMA. We found median hs-cTnI values of 39.5 ng/L in the entire cohort that were higher compared to the reports of Caselli et al. in 24 and 36 healthy neonates (21.54 ng/L and 9.3 ng/L, respectively) (9, 10). However, in our cohort, different immunoassays were used for hs-cTnI determination at the participating sites. In the majority of our SMA patients, hs-cTnI levels were measured with the Siemens/Attelica immunoassay. Because the test-specific URL for this immunoassay refers to adults, we used hs-cTnI levels obtained from neonates without SMA or cardiac disease for comparison. In both groups, similar levels of hs-cTnI were found and several neonates had levels above the test-specific URL. In their cohorts of healthy children, Caselli et al. reported the highest hs-cTnI concentrations in neonates and observed a continuous decline with increasing age (9, 10). The authors hypothesized that a rapid turnover of cardiomyocytes might explain higher neonatal levels while others discussed transient hypoxia at birth as the underlying cause (12, 13). In none of our patients with hs-cTnI levels above URL perinatal asphyxia was reported. Furthermore, we found no relevant association or correlation between the level of hs-cTnI concentrations and any of the clinical variables or CK and NT-proBNP levels in the newborns with SMA. Especially, hs-cTnI levels were not influenced by *SMN2* copy number, clinical signs of SMA and motor function score.

In one female patient with SMA and 3 *SMN2* copies hs-TnI levels (1205 and 1526 ng/L) far exceeded the upper limits measured in all other patients. Finally, the girl was diagnosed with congenital heart defect namely ASD II with left-to-right shunt and small VSD. The influence of congenital heart defects on hs-cTnI levels is still under discussion as recent studies using high-sensitive cTnI assays are also limited. Gaafar et al. reported high levels of hs-cTnI with a range of 1.7–237.0 ng/ml in children (older than 3 months of age) with cardiac defects with left-to-right shunt (14). Sugimoto et al. found higher median hs-cTnI levels in children (beyond the age of 2 months) with ASD (0.002 ng/ml) and VSD (0.029 ng/ml) compared to healthy children. The authors discussed both volume and pressure overload due to left-to-right shunt as cause for myocardial injury with subsequent elevation of hs-cTnI (15).

To date, there is no data on hs-cTnI levels in SMA patients, but there is limited data on hs-cTnT levels in these patients. Ille et al. found elevated hs-cTnT concentrations in 16 children with SMA type 1, 2 and 3, respectively and highest hs-cTnT values were measured in SMA type 1 patients (16). Du Fay et al. (17) reported that an increase in cTnT but not cTnI levels is common in adult patients with muscle disease but without cardiac involvement. The authors hypothesize that increased levels might be due to re-expression of cTnT in skeletal muscle and may also explain the data of Ille et al. who found elevated cTnT but not cTnI levels in their SMA patients (regardless of disease severity) (17). Consistent with these data there was no correlation between hs-cTnI levels and symptoms of SMA in our patients. Furthermore, hs-cTnI levels and their distribution in our cohort of neonates without SMA were similar to those in neonates with SMA, suggesting that the higher cTnI levels in our SMA cohort are primarily due to neonatal age rather than neuromuscular disease.

The use of different immunoassays in the participating centers with test-specific upper reference values obtained in adults is a daily challenge in clinical routine and was therefore also a limitation of the present study.

This is the first report of hs-cTnI levels in newborns with genetically confirmed 5q-SMA. Our results suggest higher hs-cTnI plasma levels in newborns with and without SMA compared to assay-specific reference values generated in adults. We could not identify disease-associated parameters significantly influencing hs-cTnI levels in SMA patients but one patient with a congenital heart defect showed considerably higher values compared with those of the other neonates. Due to the large obviously age-appropriate distribution of values, pre-therapeutic measurement of hs-cTnI is especially important for the interpretation of the course of post-treatment hs-cTnI values since variation from the initial values might be more significant indicating a therapy-associated myocardial affection than absolute values. Thus, as hs-cTnI levels are increasingly used in clinical routine instead of hs-cTnT levels, these data may help clinicians in the assessment of hs-cTnI levels in neonates with SMA.

## Data Availability

The datasets presented in this article are not readily available because the original contributions presented in the study are included in the article; further inquiries can be directed to the corresponding author. Requests to access the datasets should be directed to Jessika Johannsen, j.johannsen@uke.de.
