# Supplementary material for: High-sensitive cardiac troponin I (hs-cTnI) concentrations in newborns diagnosed with spinal muscular atrophy
Source: Front Pediatr. 2023 Nov 16;11:1259293. doi: 10.3389/fped.2023.1259293 (PMC10687177; doi:10.3389/fped.2023.1259293)
Supplement: Supplementary file 1 [file Table1.docx]

Table 1 (supplement): Demographic and clinical characteristics of 17 newborns with SMA and 16 newborns without SMA. Levels of hs-cTnI were determined in both groups using the Siemens/Attelica immunoassay.

*In a newborn with SMA and congenital heart disease, hs-cTnI levels (1205 ng/l) were well above those seen in other SMA patients (maximum value 234 ng/l). Median hs-cTnI level in the remaining 16 neonates with SMA but without cardiac disease was 43.5 ng/l.

# Test-specific URL: 45 ng/l, obtained in adults.

|  | newborns with SMA (n=17) | newborns without SMA (n=16) |
| --- | --- | --- |
| Gender, n (%)  male  female | 6 (35,3)  11 (64,7) | 9 (56,3)  7 (43,7) |
| Mode of delivery, n (%)  Caesarean section  vaginal delivery  unavailable data | 8 (47,1)  8 (47,1)  1 | 4 (25)  11 (68,8)  1 |
| Gestational age, n (%)  term  late-preterm  unavailable data | 14 (82,4)  3 (17,6)  0 | 14 (87,6)  1 (6,2)  1 |
| APGAR score < 8, n (%)  Yes  No  unavailable data | 1 (5,9)  15 (88,2)  1 | 2 (12,5)  11 (68,8)  3 |
| UA-pH < 7,2, n (%)  Yes  No  unavailable data | 2 (11,7)  12 (70,6)  3 | 7 (43,7)  6 (37,5)  3 |
| Assistant ventilation, n (%)  Yes  No  unavailable data | 0 (0)  17 (100)  0 | 0 (0)  16 (100)  0 |
| Feeding support, n (%)  Yes  No  unavailable data | 0 (0)  17 (100)  0 | 0 (0)  16 (100)  0 |
| Hs-cTnI levels, in ng/l ^#^  median (range) | 47 (22-234 (1205*)) | 33.5 (7-234) |
